# Supplementary material for: Molecule-based microelectromechanical sensors
Source: Sci Rep. 2018 May 22;8:8016. doi: 10.1038/s41598-018-26076-2 (PMC5964152; doi:10.1038/s41598-018-26076-2)
Supplement: Supplementary file 1 — Supplementary Information [file 41598_2018_26076_MOESM1_ESM.pdf]

Molecule-based microelectromechanical sensors

Matias Urdampilleta,* Cedric Ayela,* Pierre-Henri Ducrot, Daniel Rosario-Amorin, Abhishake Mondal,[1,2] Mathieu Rouzières, Pierre Dechambenoit, Corine Mathonière, Fabrice Mathieu, Isabelle Dufour, and Rodolphe Clérac*

Supporting Online Material

Additional experimental information.

Detailed calculation of [Δf_0/f_0]^{Material}.

The table below gathers the different mechanical parameters, which have been used in the high spin and low spin state for a very similar compound to **SCO1**[18]:

	High Spin	Low Spin	Relative variation
Young modulus (GPa)	4.77	5.16	+ 8.2 %
Mass density (kg/m^3)	1398	1471	+ 5.2 %
Poisson	0.3	0.3	0.0 %
Volume (nm^3)	2689.2	2530.5	− 5.9 %

The parameter variations at the magnetic switching change the mechanical behavior of the piezoelectric resonator as $f_0 = \frac{\lambda^2 h}{2\pi L^2}\sqrt{\frac{E}{12\rho}}$, where λ is the eigenvalue (1.875) of the first mode, h is the thickness of the resonator, L its length, E the Young's modulus and ρ the mass density. The following device parameters need to be taken into considerations:

Length L (m)	2×10^{-3}
Width b (m)	1×10^{-3}
Height h (m)	35.6×10^{-6}
Thickness of SCO h_{SCO} (m)	4×10^{-6}
**Equivalent Young modulus, E_{eq} (Pa)**	**4.9×10^{9}**
**Equivalent mass density, ρ_{eq} (kg/m^3)**	**1365**
Poisson constant, ν	0.4

The above equivalent Young's modulus and mass density correspond to the whole layered structure (PEN+Al+PVDF-TrFE+PDMS) using:

	Thickness (m)	Mass density (kg/m^3)	Young's modulus (Pa)

PEN	25×10^{-6}	1360	5×10^{9}
Al	0.6×10^{-6}	2700	69×10^{9}
PVDF-TrFE	4×10^{-6}	1800	2×10^{9}
PDMS	6×10^{-6}	965	1.8×10^{6}

and the following equations:

$$\rho_{eq} = \frac{\sum_i \rho_i h_i}{\sum_i h_i} \quad \text{and} \quad E_{eq} = \frac{4\pi^2 f_0^2 L^4 \times 12\rho_{eq}}{\lambda_0^4 h^2}$$

Finally, when we take into account the change of Young's modulus and mass density on the resonance frequency at the spin crossover temperature, we obtain:

$$\left[\frac{\Delta f_0}{f_0}\right]^{Material} = +0.16\ \%$$

Synthesis and characterization of [Fe(dmbpy)($H_2B(pz)_2$)$_2$] (SCO1): To a solution of $KH_2B(pz)_2$ (200 mg, 1.07 mmol) in MeOH (4 mL) was added $Fe(ClO_4)_2$•$6H_2O$ (195 mg, 0.54 mmol). The mixture was stirred for 10 minutes and the precipitate of potassium perchlorate was removed by filtration. A solution of 4,4'-dimethyl-2,2'-bipyridine (99 mg, 0.54 mmol) in MeOH (2 mL) was added dropwise to the colorless methanolic solution of [Fe($H_2B(pz)_2$)$_2$] and the mixture was allowed to stir for one hour. After filtration, the dark violet powder is dissolved in a CH_2Cl_2/MeOH mixture (9 mL, 2:1) and the clear solution is slowly evaporated. After one week, the resulting violet needle-shape single crystals were filtered and washed with cold methanol: Yield 185 mg (64 %). Elemental analysis Calc. $C_{24}H_{28}N_{10}FeB_2$: C, 53.97 (53.56); H, 5.28 (5.29); N, 26.23 (26.21). Selected FT-IR data (ATR, cm^{-1}): 2394 (s), 2288 (s), 1601 (s), 1558 (w), 1498 (m), 1397 (s), 1201 (m), 1155 (vs), 1051 (s), 1014 (w), 974 (s), 877 (s), 823 (s), 771 (vs), 717 (m), 639 (s). Crystallographic data are given in Table S1 as well as an ORTEP view of **SCO1** at 100 K in Figure S1. Magnetic properties of a bulk **SCO1** sample are shown in Figure S2.

Synthesis and characterization of Fe(MeOL-mCl)$_2$ (SCO2): N'-((5-chloropyridin-2-yl)methylene)-4-methoxybenzohydrazide (100 mg, 0.34 mmol), trimethylamine (100 µL, 1.36 mmol) and $Fe(ClO_4)_2$•$6H_2O$ (62 mg, 0.17 mmol) were combined in methanol (8 mL) and the mixture was stirred at room temperature for 1 hour. The resulting olive-green powder of [Fe(MeOL-mCl)$_2$] was collected by filtration and washed with methanol: Yield 89 mg (81 %). Elemental analysis Calc. (Found) for $C_{28}H_{22}Cl_2FeN_6O_4$: C, 53.11 (52.32); N, 13.27 (12.98), H, 3.50 (3.69). Selected FT-IR data (ATR, cm^{-1}): 1600 (s), 1577 (s), 1508 (m), 1447 (s), 1405 (m), 1335 (vs), 1302 (s), 1287 (s), 1248 (vs), 1159 (vs), 1141 (m), 1120 (m), 1099 (m), 1059 (s),

1022 (s), 917 (m), 906 (m), 865 (m), 839 (s), 764 (vs), 697 (s), 622 (s). Single-crystals of [Fe(MeOL-mCl)$_2$] were obtained by slow diffusion of MeOH (6 mL) into a solution of complex in CH_2Cl_2 (1.5 mL). Crystallographic data are given in Table S2 as well as an ORTEP view of **SCO2** at 120 K in Figure S3. Magnetic properties of a bulk **SCO2** sample are shown in Figure S4.

Synthesis and characterization of {[(pzTp)Fe(CN)$_3$]$_4$[Co(pz)$_3$CCH$_2$OH]$_4$[ClO$_4$]$_4$}-·13DMF·4H$_2$O (ET3): The synthesis was adapted from the one published in reference 23. Treatment of Co(ClO$_4$)$_2$•6H$_2$O (0.366 g, 1.00 mmol) in DMF (10 mL) with [NBu$_4$][(pzTp)Fe^{III}(CN)$_3$] (pzTp = tetrakis(pyrazolyl)borate 0.660 g, 1.00 mmol) afforded a dark red solution that was magnetically stirred for two hours. Addition of Et_2O (60 mL) precipitated a red oil and the supernatant was decanted; the red oil was washed with Et_2O (20 mL) and evacuated to dryness affording a red powder. The red solid was extracted into CH_2Cl_2 (15 mL) and filtered; (pz)$_3$CCH$_2$OH (0.244 g, 1.0 mmol; (pz)$_3$CCH$_2$OH = 2,2,2-*tris(1-pyrazolyl)ethanol*) was added, and the red mixture was allowed to stir for 2 hours. The solution was evacuated to dryness at room temperature. The red residue was dissolved into DMF (8 mL) and divided into two parts. Each part was layered with Et_2O (15 mL), and red block type crystals were collected after 4 days. Yield: 0.65 g (61 %). All the physical characterizations (IR, X-ray structure, magnetic properties and EA) were found to be identical to the data reported in reference 23. It is worth mentioning that the cyanido stretching absorption observed by IR spectroscopy for **ET3** (2168 cm^{-1}) remains almost the same after depositing the compound on the MEMS device (2164 cm^{-1}). A view of the **ET3** molecular structure is shown in Figure S5.

Magnetic properties: The magnetic measurements were carried out with the use of Quantum Design MPMS-XL SQUID magnetometer. These instruments work between 1.8 and 400 K with applied dc fields ranging from − 7 to 7 T. Measurements were performed on a polycrystalline sample of **SCO1** (12.95 mg) and **SCO2** (18.92 mg) sealed in a polyethylene bag (3 × 0.5 × 0.02 cm; typical 20 to 40 mg). Prior to the experiments, the field-dependent magnetization was measured at 100 K in order to confirm the absence of any bulk ferromagnetic impurities. The magnetic data were corrected for the sample holder and the intrinsic diamagnetic contributions.

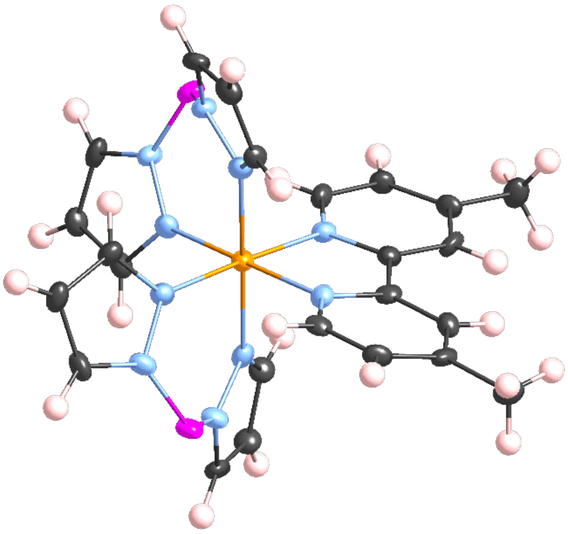

Figure S1. ORTEP view of [Fe(dmbpy)($H_2B(pz)_2)_2$] (**SCO1**) at 100 K. Thermal ellipsoids are depicted at 50 % probability. Fe orange, C grey, N blue, B pink, H light pink.

Table S1. Crystallographic data for [Fe(dmbpy)($H_2B(Pz)_2)_2$] (**SCO1**).

**Temperature, K**	100 (low-spin)	250 (high-spin)
**Crystal description**	Turquoise needle	Violet needle
**Moiety formula**	$C_{24}H_{28}FeB_2N_{10}$	$C_{24}H_{28}FeB_2N_{10}$
**Empirical formula**	$C_{24}H_{28}FeB_2N_{10}$	$C_{24}H_{28}FeB_2N_{10}$
**Formula weight**	534.01	534.01
**Crystal system**	Monoclinic	Monoclinic
**Space group**	$P2_1/c$	$P2_1/c$
**Wavelength, Å**	0.71073	0.71073
***a*, Å**	11.0414(10)	11.0705(17)
***b*, Å**	14.2520(15)	14.626(3)
***c*, Å**	19.3138(14)	19.889(3)
**β, °**	123.633(4)	123.379(8)
**V, Å^3**	2530.5(4)	2689.2(8)
Z	4	4
**ρ_{calcd}, g.cm^{-3}**	1.402	1.319
**$\mu_{MoK\alpha}$, mm^{-1}**	0.631	0.594
**R_1^{a}**	0.0503	0.0427
**wR_2^{b}**	0.1087	0.1049
**GoF^c**	1.024	1.043
**Fe-N$_{av}$, Å**	1.996	2.186
**$\Sigma_{(N\text{-}Fe\text{-}N)}$,^{d} °**	38.21	48.2

[a] $I > 2^a$ $R_1 = \sum||F_o| - |F_c||/\sum|F_o|$, [b] $wR_2 = [\sum w(F_o^2 - F_c^2)^2/\sum w(F_o^2)^2]^{1/2}$, [c] GoF (goodness of fit on F^2) $= \{\Sigma[w(F_o^2 - F_c^2)^2]/(n\text{-}p)\}^{1/2}$, where n is the number of reflections and p is the total number of refined parameters. [d] $\sum = \sum_{i=1}^{12}|90 - \varphi_i|$, where φ_i are cis N-Fe-N bond angles.

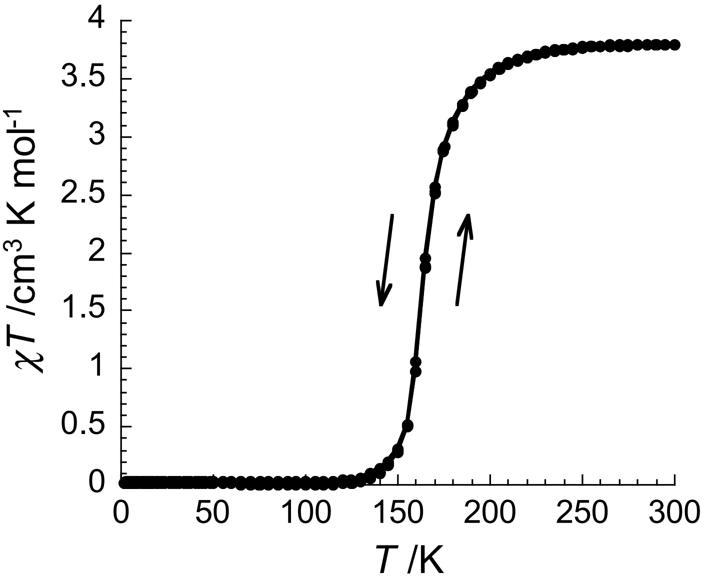

Figure S2. Temperature dependence of the χT product (χ is defined as magnetic susceptibility equal to M/H per mole of [Fe(dmbpy)($H_2B(Pz)_2)_2$], **SCO1** and T the temperature). Data collected at 0.1 and 1 T are identical in cooling and heating mode respectively (no significant thermal hysteresis effect), and thus they have been superposed on the figure. The solid lines are guides for the eyes.

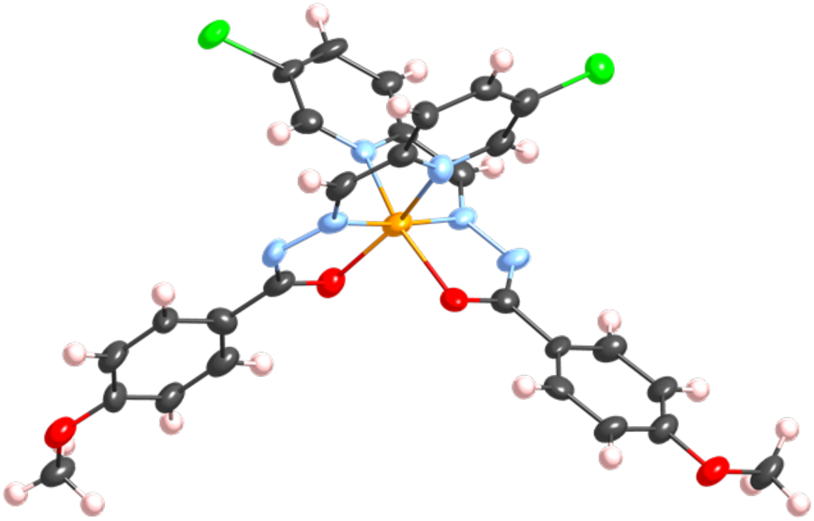

Figure S3. ORTEP view of [Fe(MeOL-mCl)$_2$] (**SCO2**) at 120 K. Thermal ellipsoids are depicted at 50 % probability. Fe orange, C grey, N blue, O red, Cl green, H light pink.

Table S2. Crystallographic data for $[Fe(MeOL\text{-}mCl)_2]$ (**SCO2**).

**Temperature, K**	120 (low-spin)	250 (high-spin)
**Crystal description**	Dark green plate	Dark green plate
**Moiety formula**	$C_{28}H_{22}Cl_2FeN_6O_4$	$C_{28}H_{22}Cl_2FeN_6O_4$
**Empirical formula**	$C_{28}H_{22}Cl_2FeN_6O_4$	$C_{28}H_{22}Cl_2FeN_6O_4$
**Formula weight**	633.26	633.26
**Crystal system**	Triclinic	Triclinic
**Space group**	*P-1*	*P-1*
**Wavelength, Å**	0.71073	0.71073
***a*, Å**	10.9324(19)	11.2203(15)
***b*, Å**	11.4057(19)	11.0521(14)
***c*, Å**	12.434(2)	12.4511(18)
α, °	106.379(8)	102.940(6)
β, °	112.323(6)	113.914(5)
γ, °	96.457(8)	94.326(5)
***V*, Å^3**	1333.2(4)	1351.5(3)
Z	2	2
ρ_{calcd}, g.cm^{-3}	1.577	1.556
$\mu_{MoK\alpha}$, mm^{-1}	0.814	0.803
R_1[a]	0.0635	0.0549
wR_2[b]	0.1523	0.1337
GoF[c]	1.003	1.015
**Fe-N$_{av}$, Å**	1.910	2.163
**Fe-O$_{av}$, Å**	2.012	2.075
$\Sigma_{(X\text{-}Fe\text{-}X)}$,[d] °	96.5	163.8

[a] $I > 2\sigma$ $R_1 = \sum||F_o| - |F_c||/\sum|F_o|$, [b] $wR_2 = [\sum w(F_o^2 - F_c^2)^2/\sum w(F_o^2)^2]^{1/2}$, [c] *GoF* (goodness of fit on F^2) = $\{\Sigma[w(F_o^2 - F_c^2)^2]/(n-p)\}^{1/2}$, where n is the number of reflections and p is the total number of refined parameters. [d] $\sum = \sum_{i=1}^{12}|90 - \varphi_i|$, where φ_i are cis X-Fe-X bond angles.

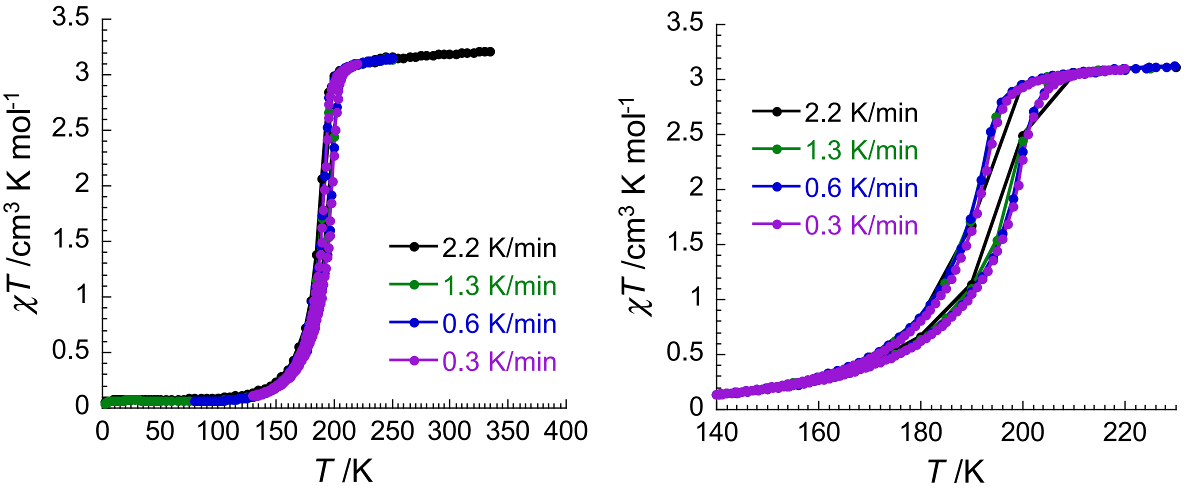

Figure S4. Temperature dependence of the χT product (χ is defined as the magnetic susceptibility equal to M/H per mole of [Fe(MeOL-mCl)$_2$], **SCO2**). Data collected at 0.1 or 1 T are identical, and thus they have been superposed on the figure. The solid lines are guides for the eyes.

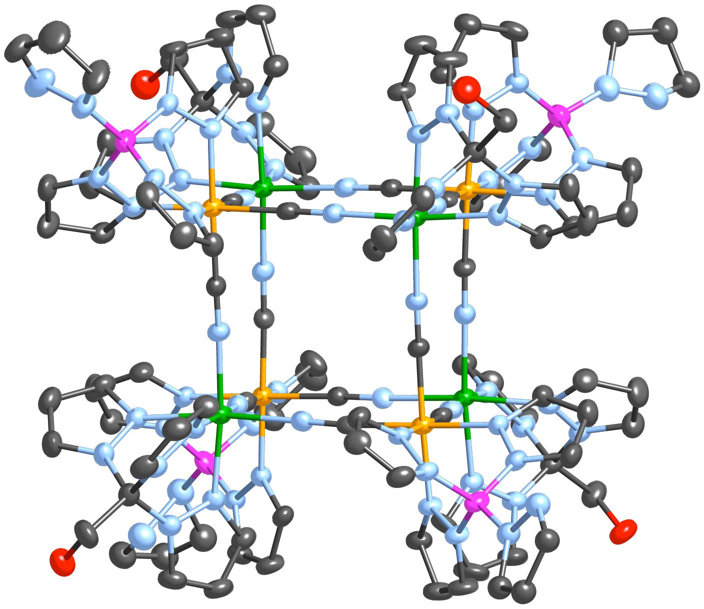

Figure S5. ORTEP view of **{[(pzTp)Fe^{III}(CN)$_3$]$_4$[Co^{II}(pz)$_3$CCH$_2$OH]$_4$[ClO$_4$]$_4$}-·13DMF·4H$_2$O (ET3)** at 90 K from reference 23. Thermal ellipsoids are depicted at 20 % probability. Fe orange, Co green, C grey, N blue, O red, B pink. The magnetic properties of **ET3** can be found in reference 23.
